# Suzetrigine (a NaV1.8 inhibitor) versus placebo for acute postoperative pain: A systematic review and meta-analysis of randomized controlled trials

**DOI:** 10.1097/MD.0000000000047877

**Published:** 2026-03-06

**Authors:** Pablo Alvarez-Aguilar, Susimar Picado-Loaiza

**Affiliations:** a Department of Critical Care, Hospital México, Caja Costarricense del Seguro Social, San José, Costa Rica; b Physiology Department, School of Medicine, University of Costa Rica, San Pedro, San José, Costa Rica; c Department of Cardiovascular Surgery, Hospital México, Caja Costarricense del Seguro Social, San José, Costa Rica.

**Keywords:** NaV1.8, NaV1.8 inhibitor, postoperative pain, suzetrigine, VX-548

## Abstract

**Background:**

NaV1.8 channels, expressed in peripheral nociceptors, mediate sustained pain signaling. Their inhibition offers a potential opioid-sparing strategy for postoperative pain, although efficacy and safety remain incompletely defined. We synthesized randomized evidence for suzetrigine versus placebo in postoperative pain.

**Methods:**

We conducted a Preferred Reporting Items for Systematic Reviews and Meta-Analyses-compliant meta-analysis of randomized controlled trials comparing suzetrigine (a NaV1.8 inhibitor) versus placebo in surgical patients. The primary outcome was 24-hour pain with the Numeric Pain Rating Scale; secondary outcomes included 48-hour pain, change-from-baseline, and adverse events. Risk of bias was assessed with the Cochrane Risk of Bias 2 tool, and for analyses, we used Hartung–Knapp models with heterogeneity and prediction intervals reported. Random-effects models used Hartung–Knapp adjustments (2-sided α = 0.05).

**Results:**

We included 4 randomized datasets across 2 phase 3 publications (n = 1584; 1009 intervention, 575 placebo). The mean participant age was 44.9 years; 92.3% were women. Procedures included abdominoplasty (n = 823) and bunionectomy (n = 761). Suzetrigine significantly reduced pain at 24 hours (mean difference = −0.93; 95% confidence interval [CI], −1.38 to −0.48; *I*^2^ = 66.0%) and 48 hours (mean difference = −1.02; 95% CI, −1.32 to −0.72; *I*^2^ = 11.8%). Analyses of change-from-baseline confirmed consistent benefit. Subgroup analyses revealed similar effects across surgery types. A lower incidence of nausea (risk ratio = 0.63; 95% CI, 0.42–0.95) and dizziness (risk ratio = 0.57; 95% CI, 0.34–0.96) was observed in the suzetrigine group, with no significant differences in headache, vomiting, or constipation. Meta-regressions showed no moderation by sample size or publication year. Risk of bias was low in 2 studies and raised “some concerns” in 2.

**Conclusion:**

Suzetrigine produced modest reductions in pain at 24 to 48 hours versus placebo. Because opioid consumption was not consistently reported, no conclusions can be drawn regarding opioid-sparing.

## 1. Introduction

Acute postoperative pain remains a major clinical issue, and despite multimodal regimens, many patients endure moderate-to-severe pain during the first 48 hours after surgery, which delays recovery, increases the risk of complications, and predisposes patients to chronic pain.^[1,2]^ Pain persistence reflects patient comorbidities, surgical invasiveness, and suboptimal analgesic efficacy, and diminishes the patient’s immediate postoperative experience but also sets the stage for long-term sequelae that can significantly impact their overall quality of life.^[3]^

NaV1.8, a voltage-gated sodium channel on peripheral nociceptors, represents a selective analgesic target, and genetic ablation and pharmacologic blockade both attenuate nociceptive transmission in preclinical models without central opioid-related side effects, supporting NaV1.8 inhibitors as valuable adjuncts in multimodal postoperative pain protocols.^[4]^ NaV1.8 sodium channel blockade has emerged as a promising adjunct in multimodal analgesic protocols for postoperative pain management.^[5]^

To clarify their risk–benefit balance, we conducted a systematic review and meta-analysis assessing the analgesic effectiveness and adverse events associated with the NaV1.8 blockade with suzetrigine after surgery.

## 2. Materials and methods

This systematic review and meta-analysis was conducted and reported in accordance with the Cochrane Handbook for Systematic Review of Interventions and the Preferred Reporting Items for Systematic Reviews and Meta-Analyses guidelines.^[6,7]^ The prospective meta-analysis protocol was registered in the International Prospective Register of Systematic Reviews on May 21, 2025 (registration number CRD420251057157).

### 2.1. Eligibility criteria

Inclusion criteria were restricted to studies that met all of the following: adult patients aged ≥18 years with no restriction on sex or ethnicity undergoing any type of elective or emergency surgical intervention, performed under general, regional, or combined anesthesia, experiencing acute postoperative pain; comparing of NaV1.8 blockade with suzetrigine *at optimal clinical dose* versus placebo; reported at least one of the following outcomes: postoperative pain intensity using the Numeric Pain Rating Scale (NPRS) at 24 or 48 hours, and shared clinical outcomes; provided sufficient numerical data to extract or calculate mean differences (MDs; for continuous outcomes) or risk ratios (RRs; for binary outcomes); and randomized controlled trials (RCTs). We excluded studies with pediatric or nonadult populations; chronic or nonsurgical pain conditions; emergency-only settings; combined interventions without isolatable data; single-arm studies; subtherapeutic dose regimen (identified in preclinical studies); non-placebo-controlled or nonrandomized designs; insufficient or incompatible outcome reporting (as trials without extractable NPRS data); and abstracts, conference proceedings, or reports for which full numeric data could not be obtained.

### 2.2. Search strategy and data extraction

We systematically searched PubMed, Embase, Latin American and Caribbean Health Sciences Literature, and the Cochrane Central Register of Controlled Trials; searches were performed on May 21, 2025, using the following terms across all databases: (“postoperative pain”) AND (“NaV1.8” OR “NaV1.8 inhibitor” OR “NaV1.8 antagonist” OR “tetrodotoxin-resistant sodium channel blocker” OR “VX-548” OR “suzetrigine” OR “A-803467” OR “Journavx”).

References from all included studies were also searched manually. Two authors (P.A. and S.P.) independently extracted the data following the predefined search criteria and quality assessment. The detailed search strategy is described in Supplementary Methods, https://links.lww.com/MD/R474.

### 2.3. Outcomes

The primary outcome was pain intensity at 24 hours postoperatively (NPRS). Secondary outcomes included NPRS at 48 hours; reduction of pain intensity according to the NPRS at 24 and 48 hours; and incidence of dizziness, nausea, headache, vomiting, and constipation. Pain outcomes were treated as continuous variables (MD), and adverse events as binary variables (RR).

### 2.4. Statistical analysis

Data were extracted from the study tables by 2 authors using standard digitization techniques through dedicated spreadsheets. When pain scores at 24 or 48 hours were not available in tabular form, data were extracted from the published figures using WebPlotDigitizer (version 4.6; Automeris LLC, Pacifica), a validated graphical data extraction tool. Two authors independently extracted and verified all numeric values. All meta-analyses were conducted in R (version 4.3.2; R Foundation for Statistical Computing, Vienna, Austria) using the meta-, metafor-, ggplot2-, and dplyr-based packages. All tests were 2-sided (α = 0.05). Confidence intervals (CIs) used Hartung–Knapp adjustments.

### 2.5. Meta-analyses

Continuous outcomes were analyzed using inverse variance random-effects models via the Hartung–Knapp–Sidik–Jonkman method for CIs.^[8]^ Binary outcomes were pooled using random-effects RRs with the Hartung–Knapp–Sidik–Jonkman correction and DerSimonian–Laird estimator for τ^2^. For each outcome, we calculated 95% CI, between-study heterogeneity (*I*^2^ and τ^2^), and 95% prediction intervals. Forest plots were generated for all the pooled outcomes. All plots were exported at high-resolution .jpg and .tiff files.

### 2.6. Subgroup analyses

We conducted subgroup analyses according to surgery type (abdominoplasty vs bunionectomy) for all outcomes. Subgroup-specific pooled estimates were reported along with heterogeneity within and between subgroups. The tests for subgroup differences were evaluated using *Q* statistics.

### 2.7. Sensitivity analysis

We performed leave-one-out sensitivity analyses for all outcomes. For each analysis, we recalculated the pooled effects by iteratively omitting 1 study at a time.

### 2.8. Meta-regression

Random-effects meta-regressions were performed for continuous and binary outcomes to explore the potential impact of publication year and sample size on the effect estimates. Moderators were treated as continuous variables (standardized total sample size [*z*-score] and numeric years). Bubble plots were created for each outcome, showing the regression line and study weight (inverse variance).

### 2.9. Software and reproducibility

All analyses were performed in R. All plots, regression models, and forest/funnel/bubble visualizations were scripted reproducibly. A complete analysis script and an output archive are available upon request.

### 2.10. Quality assessment

Two independent authors (P.A. and S.P.) completed the risk of bias assessment in randomized studies using version 2 of the Cochrane Risk of Bias assessment tool.^[9]^ Disagreements were resolved through consensus after discussing the reasons for the discrepancy.

Publication bias was investigated using funnel plot analysis of point estimates in relation to the study weights. Although we assessed risk of bias using the Cochrane Risk of Bias 2.0 tool (Cochrane, London, United Kingdom), we did not conduct a formal Grading of Recommendations Assessment, Development and Evaluation assessment of the certainty of evidence. However, based on the small number of included studies, some concerns of bias, and limited subgroup power, the overall certainty for most outcomes would likely be moderate to low. We recommend cautious interpretation until more independent trials become available.

## 3. Results

### 3.1. Study selection and characteristics

**Figure 1.**
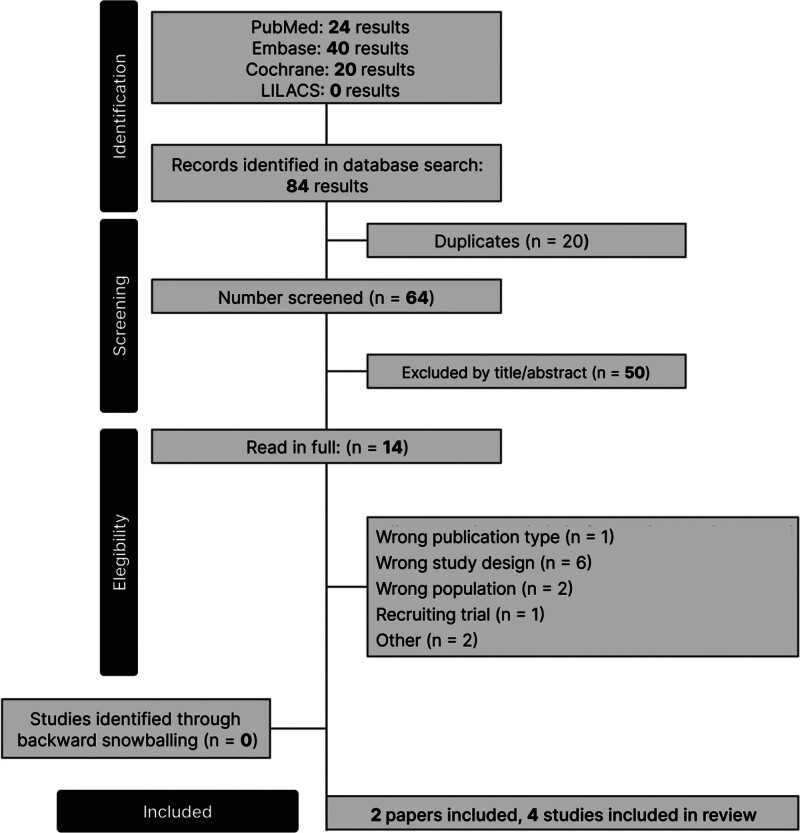
PRISMA flow diagram of study identification, screening, eligibility assessment, and inclusion for the systematic review and meta-analysis for randomized controlled trials of suzetrigine versus placebo in acute postoperative pain. LILACS = Latin American and Caribbean Health Sciences Literature, PRISMA = Preferred Reporting Items for Systematic Reviews and Meta-Analyses. Source: Page et al^[6]^

As detailed in Figure 1, the initial search yielded a total of 64 results. After screening and removal of duplicate records and ineligible studies, 2 papers with 2 studies were included, which were fully reviewed based on inclusion criteria, comprising a total of 1584 patients from 4 RCTs (1009 intervention and 575 placebo). The mean age was 44.94 ± 11.48 years, with 92.3% female patients. Abdominoplasty was performed in 823 patients, and bunionectomy in 761 patients. Baseline characteristics and intervention details of the RCTs included in the review are summarized in Tables 1 and 2.

**Table 1:** Baseline characteristics and intervention details of the randomized controlled trials included in the review comparing NaV1.8 channel blockers versus placebo for acute postoperative pain.

Study	Jones, 2023 (abdominoplasty)^[9]^	Jones, 2023 (bunionectomy)^[9]^	D’Aunno, NAVIGATE-2, 2025 (abdominoplasty)^[8]^	Bertoch, NAVIGATE-1, 2025 (bunionectomy)^[8]^
Registry	NCT05034952	NCT04977336	NCT05558410	NCT05553366
Sample size	153	119	670	642
Age	42.8 ± 9.6	47.7 ± 13.6	42.0 ± 8.7	48.0 ± 13.0
Females, No. (%)	151 (98.7)	102 (85.7)	657 (98.1)	553 (86.1)
Treatment (n)	100 mg loading dose, then 50 mg/12 h (76)	100 mg loading dose, then 50 mg/12 h (60)	100 mg loading dose, then 50 mg every 12 h (447)	100 mg loading dose, then 50 mg every 12 h (426)
Placebo (n)	77	59	223	216
Anesthetic strategy	General anesthesia with propofol and fentanyl	Regional anesthesia: Mayo block and popliteal sciatic nerve block for 1 d	General anesthesia with propofol and fentanyl	Regional anesthesia: Mayo and popliteal block with ropivacaine for 1 d

**Table 2:** Intervention details and outcomes of the randomized controlled trials included in the review comparing NaV1.8 channel blockers versus placebo for acute postoperative pain.

Study	Jones, 2023^[9]^	Jones, 2023^[9]^	D’Aunno, NAVIGATE-2, 2025^[8]^	Bertoch, NAVIGATE-1, 2025^[8]^
Analgesic strategy	Acetaminophen, fentanyl, and ropivacaine boluses and infusion. No treatments for pain were allowed after removal of the popliteal block up to the time of randomization.	Acetaminophen, fentanyl, and ropivacaine boluses and infusion. No treatments for pain were allowed after removal of the popliteal block up to the time of randomization.	Acetaminophen, fentanyl, ropivacaine boluses and/or infusion, rescue with ibuprofen, ice packs use was not permitted.	Acetaminophen, fentanyl, ropivacaine boluses and/or infusion, rescue with ibuprofen, ice packs use was not permitted.
NPRS score at rest	7.3 ± 1.65	6.80 ± 1.70	7.37 ± 1.7	6.73 ± 1.80
VRS, No. (%)				
Moderate	86 (56)	83 (70)	393 (59)	438 (68)
Severe	67 (44)	36 (30)	277 (41)	204 (32)
Primary outcomes	Primary: SPID48, LSM, LSM vs placebo	Primary: SPID48, LSM, LSM vs placebo	SPID48	SPID48
Secondary outcomes	SPID24, LSM, LSM vs placebo	SPID24, LSM, LSM vs placebo	Time to 2-point or greater reduction in NPRS from baseline	Time to 2-point or greater reduction in NPRS from baseline
Adverse effects	Nausea, headache, constipation, dizziness, vomiting	Nausea, headache, constipation, dizziness, vomiting	Nausea, headache, constipation, dizziness, vomiting, hypotension	Nausea, headache, constipation, dizziness, vomiting, hypotension

LSM = least-squares means, NPRS = Numeric Pain Rating Scale, SPID = summed pain-intensity difference (time-weighted sum), SPID48 = SPID over 48 hours, VRS = verbal categorical rating scale.

### 3.2. Pain outcomes

**Figure 2.**
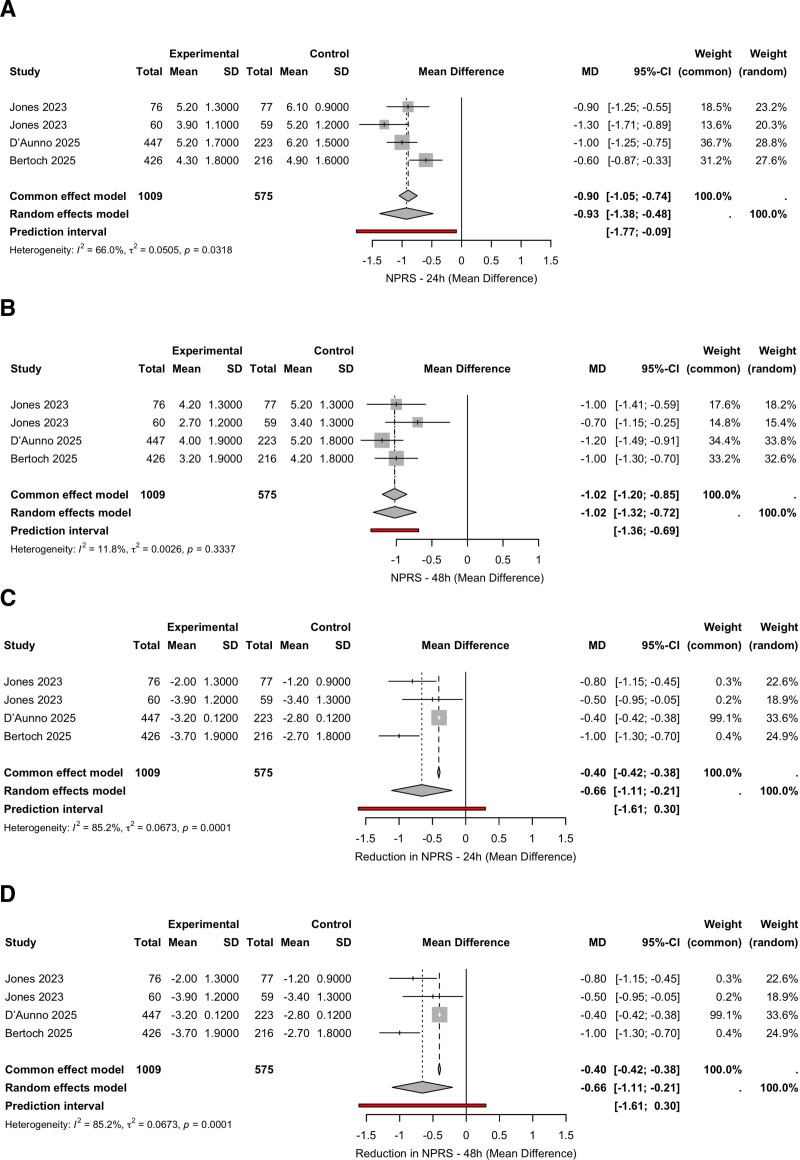
Forest plots of mean differences in pain intensity between suzetrigine and control. (A) NPRS at 24 hours. (B) NPRS at 48 hours. (C) Reduction in NPRS at 24 hours. (D) Reduction in NPRS at 48 hours. Negative values favor NaV1.8 treatment. CI = confidence interval, MD = mean difference, NPRS = Numeric Pain Rating Scale, SD = standard deviation.

Suzetrigine significantly reduced pain scores at both time points. At 24 hours, the pooled MD was −0.93 (95% CI, −1.38 to −0.48; *I*^2^ = 66.0%), with a prediction interval of −1.77 to −0.09 (Fig. 2A). At 48 hours, the MD was −1.02 (95% CI, −1.32 to −0.72; *I*^2^ = 11.8%; Fig. 2B). Analyses of change-from-baseline showed consistent findings at both 24 hours (MD = −0.66; 95% CI, −1.11 to −0.21; *I*^2^ = 85.2%; Fig. 2C) and 48 hours (MD = −0.66; 95% CI, −1.11 to −0.21; *I*^2^ = 85.2%; Fig. 2D), although prediction intervals included the null values.

### 3.3. Subgroup analyses

**Figure 3.**
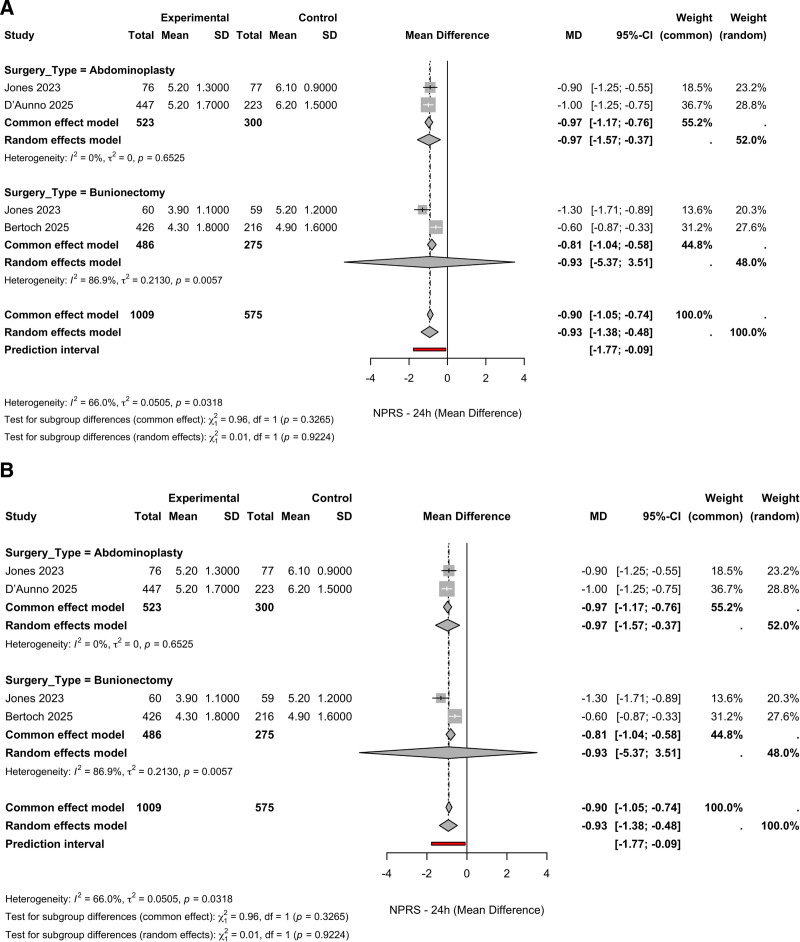
Subgroup analyses by surgery type for NPRS scores at 24 hours. (A) Forest plot showing mean differences in NPRS by surgery type (abdominoplasty vs bunionectomy). (B) Same analysis using alternate weight scaling. Negative values favor suzetrigine. CI = confidence interval, MD = mean difference, NPRS = Numeric Pain Rating Scale, SD = standard deviation.

Subgroup analysis according to surgical type (Fig. 3) showed consistent benefit. In the abdominoplasty subgroup (n = 823), the pooled MD was −0.97 (95% CI, −1.57 to −0.37; *I*^2^ = 0%). In the bunionectomy subgroup (n = 761), the MD was −0.93 (95% CI, −5.37 to 3.51; *I*^2^ = 86.9%). No significant subgroup differences were detected (*P* = .33 for the common-effect model; *P* = .92 for the random-effects model). Overall, heterogeneity was moderate, driven primarily by the bunionectomy subgroup. The elevated heterogeneity observed in the bunionectomy subgroup (*I*^2^ = 86.9%), compared with the abdominoplasty subgroup (*I*^2^ = 0%), may reflect differences in baseline pain scores, regional anesthesia techniques (e.g., Mayo vs popliteal blocks), and the availability or timing of rescue analgesia. Although both studies implemented standardized protocols, the effect of these subtle perioperative differences could not be fully disentangled in our analysis (Fig. 3A and B).

### 3.4. Adverse events

**Figure 4.**
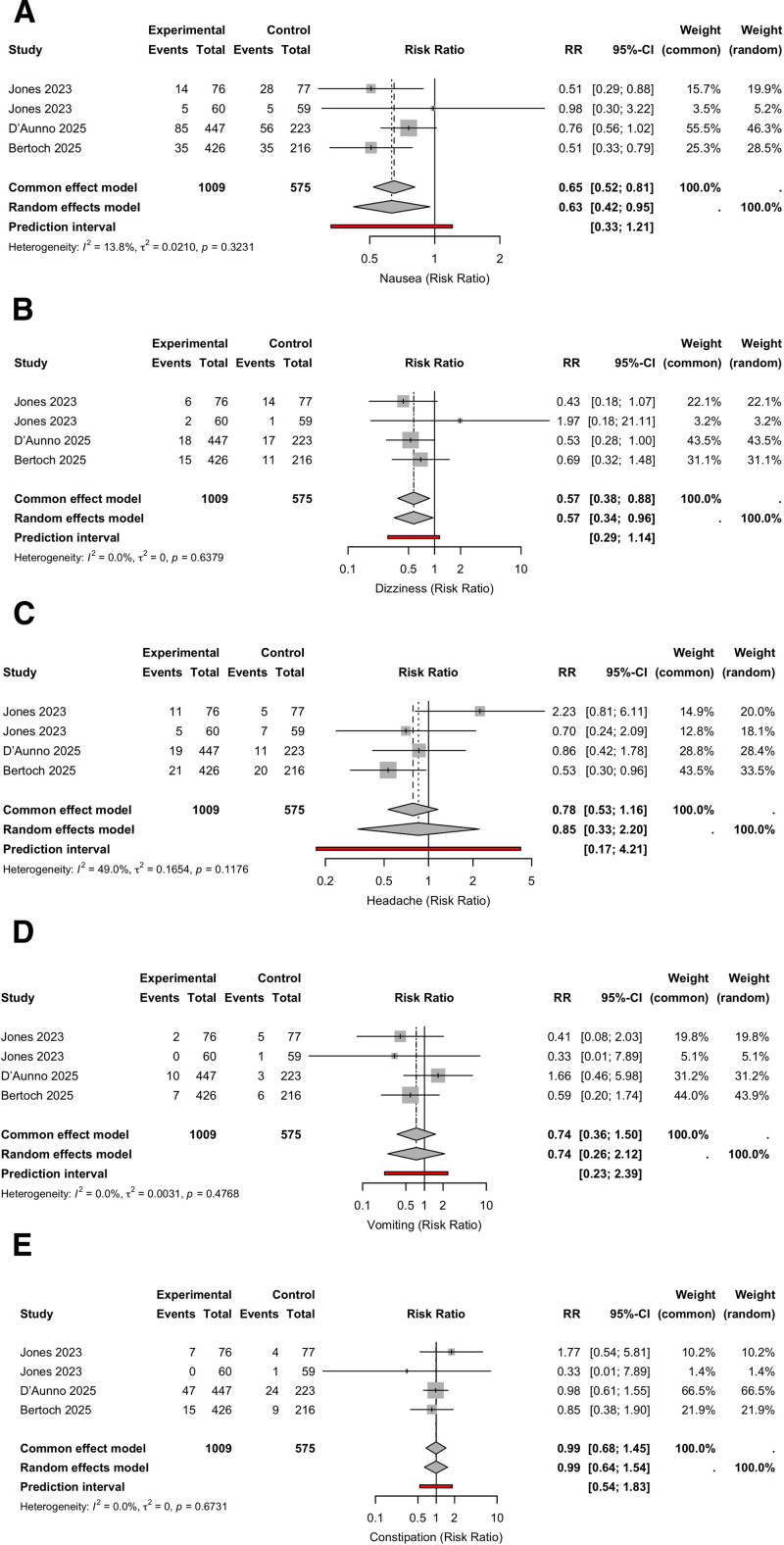
Forest plots of risk ratios for common adverse events comparing suzetrigine versus control. Negative values (RR < 1) favor the suzetrigine group. Outcomes shown include nausea (A), dizziness (B), headache (C), vomiting (D), and constipation (E). CI = confidence interval, RR = risk ratio.

Suzetrigine significantly reduced the risk of nausea (RR = 0.63; 95% CI, 0.42–0.95; *I*^2^ = 13.8%) and dizziness (RR = 0.57; 95% CI, 0.34–0.96; *I*^2^ = 0%). No significant effects were observed on vomiting (RR = 0.74; 95% CI, 0.26–2.12), headache (RR = 0.85; 95% CI, 0.33–2.20), or constipation (RR = 0.99; 95% CI, 0.64–1.54; Fig. 4).

### 3.5. Logistic regression

**Figure 5.**
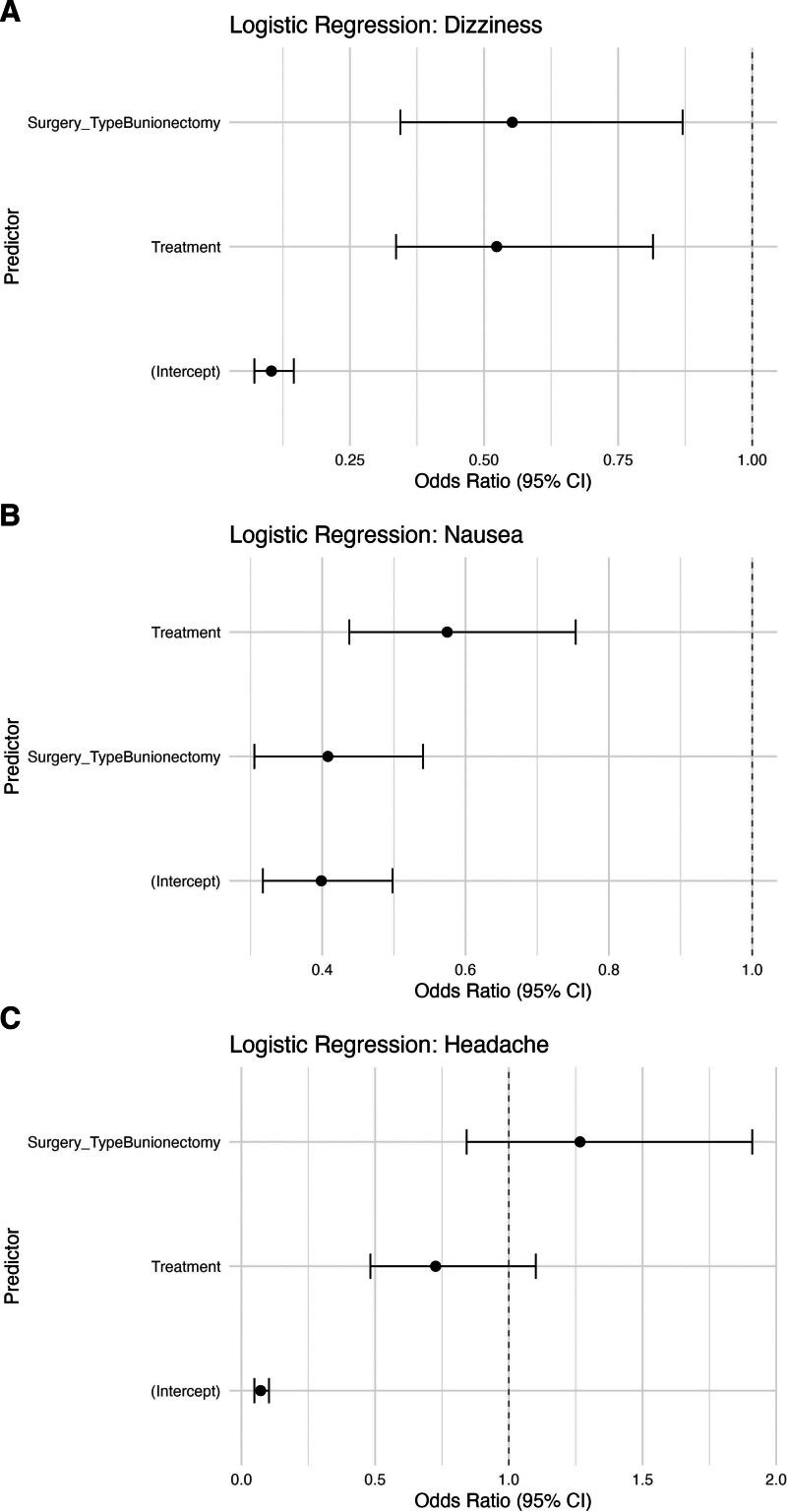
Adjusted logistic regression models for adverse events. Dot-and-whisker plots show odds ratios (points) and 95% confidence intervals (lines) for the association of treatment (suzetrigine vs placebo) and surgery type (abdominoplasty vs bunionectomy) with (A) dizziness, (B) nausea, and (C) headache. The vertical dashed line denotes the null value (OR = 1). Intercepts are shown for completeness but are not clinically interpretable. CI = confidence interval, OR = odds ratio.

Adjusted models confirmed that suzetrigine independently reduced the odds of nausea (odds ratio [OR] = 0.57; 95% CI, 0.44–0.75) and dizziness (OR = 0.52; 95% CI, 0.34–0.81). These adjusted effects are illustrated in Figure 5, which displays the dot-and-whisker plots of the ORs and 95% CIs for treatment assignment and surgery type across models for dizziness, nausea, and headache (vertical dashed line at OR = 1). Bunionectomy was also associated with lower odds of nausea (OR = 0.41) and dizziness (OR = 0.55). Because our synthesis relied on aggregate, study-level data, we did not perform patient-level adjusted logistic regression. As an exploratory approach, we conducted influence diagnostics (leave-one-out) and examined between-study heterogeneity, effect direction remained consistent, and the magnitude varied within the 95% prediction interval. Full exploratory outputs are provided in eTables/eFigures, Supplemental Digital Content, https://links.lww.com/MD/R474.

### 3.6. Meta-regression

No significant moderation by sample size or publication year was observed for any continuous or binary outcomes (see Figs. S1 and S2, Supplemental Digital Content, https://links.lww.com/MD/R474). Trends suggested marginally smaller effects in larger and more recent trials for pain, but the explained heterogeneity (*R*^2^) was negligible. Although no statistically significant moderation was observed, these analyses are inherently limited by the small number of studies. Consequently, the absence of effect modification should not be interpreted as conclusive, and further studies are needed to confirm these patterns.

### 3.7. Sensitivity analyses

Leave-one-out analyses demonstrated stable effect estimates for the NPRS at 24 and 48 hours, as well as for nausea and dizziness. No single study has influenced the results. By contrast, estimates for headache, vomiting, and constipation were inconsistent and less robust (see Figs. S3–S5, Supplemental Digital Content, https://links.lww.com/MD/R474).

### 3.8. Risk of bias and publication bias

Two studies were rated as having a low risk of bias across all domains, while 2 were assessed as having “some concerns” due to unclear randomization procedures. A summary of the risk of bias assessment is provided in Figure S6, Supplemental Digital Content, https://links.lww.com/MD/R474. Funnel plots for the primary outcome, secondary continuous outcomes, and binary outcomes are shown in Figures S7 to S9, Supplemental Digital Content, https://links.lww.com/MD/R474. Given the limited number of included studies, formal tests for funnel plot asymmetry, such as the Egger test, were not performed.

## 4. Discussion

This meta-analysis specifically evaluates suzetrigine (a selective NaV1.8 inhibitor) versus placebo in acute postoperative pain across abdominoplasty and bunionectomy datasets. Suzetrigine was associated with modest reductions in pain at 24 to 48 hours compared with placebo, with consistent directionality across the 4 randomized datasets derived from 2 phase 3 publications, supporting its clinical relevance in multimodal analgesia.^[8]^ These findings are aligned with robust preclinical and clinical evidence validating suzetrigine as a peripheral nociceptive target with minimal central nervous system (CNS) penetration.^[4,9]^

The pooled MDs indicate a modest analgesic effect. When contextualized against commonly cited minimal clinically important difference ranges for acute postoperative pain on the 0 to 10 NPRS (often ~1–2 points and population-dependent), the clinical salience is likely context-specific, varying with baseline pain intensity, co-analgesic regimens, procedure type, and patient preferences. We therefore avoid overinterpretation and present these findings as supportive but not definitive for broad clinical adoption.

NaV1.8 is a tetrodotoxin-resistant sodium channel that is selectively expressed in peripheral sensory neurons, particularly small-diameter nociceptors in the dorsal root ganglia.^[4,10]^ It contributes to action potential propagation during sustained depolarization and is key for repetitive firing in response to noxious stimuli.^[11]^ Unlike NaV1.7 or NaV1.9, NaV1.8 exhibits rapid recovery from inactivation, enabling neurons to fire at high frequencies – a property particularly relevant to pain chronification.^[12]^

The analgesic effect observed in our study (MD = −0.93 at 24 hours and −1.02 at 48 hours) mirrors the efficacy of suzetrigine in separate trials involving moderate-to-severe postoperative pain, although no direct comparison with opioids was conducted in this meta-analysis.^[9]^ Previous literature suggests that the minimal clinically important difference for acute postoperative pain on the NPRS ranges between 0.31 and 2.0 points.^[13,14]^ Therefore, the effect size observed here may fall below the threshold of perceptible benefit for many patients and should be interpreted in the context of baseline pain, multimodal strategies, and patient preferences. Notably, suzetrigine demonstrated faster onset of analgesia (e.g., median time to NPRS-2 reduction: 119 minutes vs 480 minutes after abdominoplasty) and favorable tolerability with only mild-to-moderate side effects.^[9]^

From a mechanistic standpoint, the therapeutic window for suzetrigine is favorable. It is absent from the CNS,^[15,16]^ minimizing the risk of sedation or cognitive impairment. This peripherally restricted expression makes suzetrigine an ideal target for nonopioid analgesics. Pharmacological agents such as suzetrigine, VX-150, and A-803467 exert their effect by stabilizing the inactivated state of the channel, thereby reducing afferent nociceptive transmission without impairing sensory or motor function.^[17,18]^

In our analysis, suzetrigine also significantly reduced nausea and dizziness, 2 adverse effects commonly associated with opioids. These benefits were observed across both surgical procedures analyzed, although no formal adjustment was possible due to limited data. The peripheral mechanism of suzetrigine may contribute to a favorable side effect profile, including reduced sedation and dizziness, although further research is needed to conclusively establish this.^[8,16]^

The promise of NaV1.8 inhibition extends beyond postoperative pain. Gain-of-function mutations in *SCN10A* (encoding NaV1.8) are associated with painful neuropathies and familial episodic pain, whereas loss-of-function variants confer insensitivity to pain.^[17,19]^ Preclinical models have confirmed that selective NaV1.8 inhibition reverses mechanical allodynia and thermal hyperalgesia in inflammatory and neuropathic pain.^[17]^ Importantly, degeneracy among sodium channels, whereby dorsal root ganglia neurons can maintain excitability via alternate isoforms, may explain why some patients respond better to NaV1.8 inhibition than to NaV1.7 blockade alone.^[20]^

Although VX-548 and VX-150 have shown consistent efficacy, several NaV1.7 inhibitors have failed in clinical trials despite promising preclinical profiles, possibly due to insufficient engagement or compensatory upregulation of NaV1.8 and NaV1.9.^[11,20]^ This highlights the therapeutic potential of dual or preferential targeting of NaV1.8, especially in patients with inflammatory or chronic pain phenotypes.^[19]^

Despite these encouraging findings, the limitations of the present study must be acknowledged. First, the number of eligible RCTs was small (n = 4), with all studies coming from only 2 independent publications. Although the total sample size was relatively large, this limited the power of the meta-regression, the robustness of subgroup analyses, and the detection of publication bias. Additionally, the fact that all studies were sponsored by the same pharmaceutical company raises the possibility of selective outcome reporting or industry-related bias. Opioid consumption and conventional analgesic dose-reduction were not consistently reported across the included randomized datasets and were not prespecified secondary outcomes; therefore, we draw no conclusions regarding an opioid-sparing effect of suzetrigine in this synthesis. These factors reduce the certainty and generalizability of the findings. Second, the outcomes were restricted to short-term endpoints (≤48 hours), and evidence on long-term benefits and chronic pain prevention remains sparse. Third, while the risk of bias was generally low, 2 studies had “some concerns” related to the randomization sequence, and publication bias could not be fully ruled out. Finally, different surgical types, anesthetic approaches, and rescue protocols introduce heterogeneity.

Future directions should include direct comparisons between NaV1.8 inhibitors and nonsteroidal anti-inflammatory drugs or regional anesthesia in enhanced recovery after surgery protocols. Moreover, a longer follow-up is necessary to evaluate their role in preventing chronic postoperative pain, a condition affecting up to 30% of patients following major surgery.^[2]^

In summary, the NaV1.8 blockade with suzetrigine represents a biologically rational, clinically validated strategy to reduce postoperative pain intensity and opioid-related side effects. With robust pharmacological specificity, peripheral action, and favorable safety profile, suzetrigine may serve as a cornerstone agent in future multimodal analgesia pathways.

Furthermore, a network meta-analysis was not feasible due to the absence of studies directly comparing suzetrigine with standard analgesics such as hydrocodone. Future research should address these comparisons to contextualize their clinical relevance.

A recent systematic review by Amaral et al addressed a similar question using the same phase 3 trials.^[21]^ Our analysis expands on their findings by including change-from-baseline scores and subgroup heterogeneity analyses.

Our study was limited by the inclusion of only 2 RCTs, which were further split by surgical site, effectively reducing the number of independent datasets. Additionally, both trials were sponsored by the same pharmaceutical company, potentially introducing a bias. No unpublished studies were retrieved, and we did not search ClinicalTrials.gov for ongoing trials. We also searched ClinicalTrials.gov for ongoing or unpublished trials using the same search terms and filters. No eligible unpublished studies with extractable data were identified. A phase IV open-label study (NCT06887972) was found but excluded due to its single-arm design. Finally, while adverse events such as nausea and dizziness were evaluated, no data were available on serious adverse events, cognitive side effects, or long-term safety, which limits the full characterization of the safety profile of suzetrigine in clinical use.

## 5. Conclusions

Suzetrigine demonstrates modest short-term analgesic benefits versus placebo in postoperative settings. Evidence on opioid-sparing effects, overall opioid consumption, and long-term safety remains limited, underscoring the need for independent, adequately powered randomized trials with standardized co-analgesic reporting and longer follow-up. Given its peripheral NaV1.8 blockade, minimal CNS penetration, and emerging short-term safety profile, suzetrigine and NaV1.8 inhibitors more broadly may serve as promising nonopioid components of multimodal, enhanced recovery after surgery-aligned analgesia. Further trials should assess durability of benefit, opioid-sparing potential, and prevention of chronic postsurgical pain across diverse surgical populations.

## Author contributions

**Conceptualization:** Pablo Alvarez-Aguilar.

**Data curation:** Pablo Alvarez-Aguilar, Susimar Picado-Loaiza.

**Formal analysis:** Pablo Alvarez-Aguilar, Susimar Picado-Loaiza.

**Methodology:** Pablo Alvarez-Aguilar, Susimar Picado-Loaiza.

**Project administration:** Pablo Alvarez-Aguilar.

**Supervision:** Pablo Alvarez-Aguilar.

**Investigation:** Susimar Picado-Loaiza.

**Visualization:** Susimar Picado-Loaiza.

**Validation:** Pablo Alvarez-Aguilar, Susimar Picado-Loaiza.

**Writing – original draft:** Pablo Alvarez-Aguilar, Susimar Picado-Loaiza.

**Writing – review & editing:** Pablo Alvarez-Aguilar, Susimar Picado-Loaiza.

## Supplementary Material


