# Supplementary material for: Suzetrigine (a NaV1.8 inhibitor) versus placebo for acute postoperative pain: A systematic review and meta-analysis of randomized controlled trials
Source: Medicine (Baltimore). 2026 Mar 6;105(10):e47877. doi: 10.1097/MD.0000000000047877 (PMC12975200; doi:10.1097/MD.0000000000047877)

Supplementary Material

Supplementary Figure S1: Meta-regression bubble plot for 24-hour pain outcome according to sample size.

Supplementary Figure S2: Meta-regression bubble plot for 24-hour pain outcome according to publication year.

Supplementary Figure S3: Leave-one-out sensitivity analysis for 24-hour NPRS.

Supplementary Figure S4: Leave-one-out sensitivity analysis for nausea.

Supplementary Figure S5: Leave-one-out sensitivity analysis for dizziness.

Supplementary Figure S6: Risk of bias assessment summary using RoB 2.0 tool.

Supplementary Figure S7: Funnel plot for the primary outcome (NPRS at 24h).

Supplementary Figure S8: Funnel plot for continuous secondary outcomes (NPRS at 48h, change-from-baseline).

Supplementary Figure S9: Funnel plot for binary outcomes (adverse events).

Supplementary Methods. Search strategy

(postoperative pain) AND (NaV1.8 OR NaV1.8 inhibitor OR NaV1.8 antagonist OR NaV 1.8 blocker OR tetrodotoxin-resistant sodium channel blocker OR A-803467 OR A 803467 OR VX-548 OR suzetrigine OR Journavx)

Figure S1 – Meta regression: bubble plot continuous outcomes

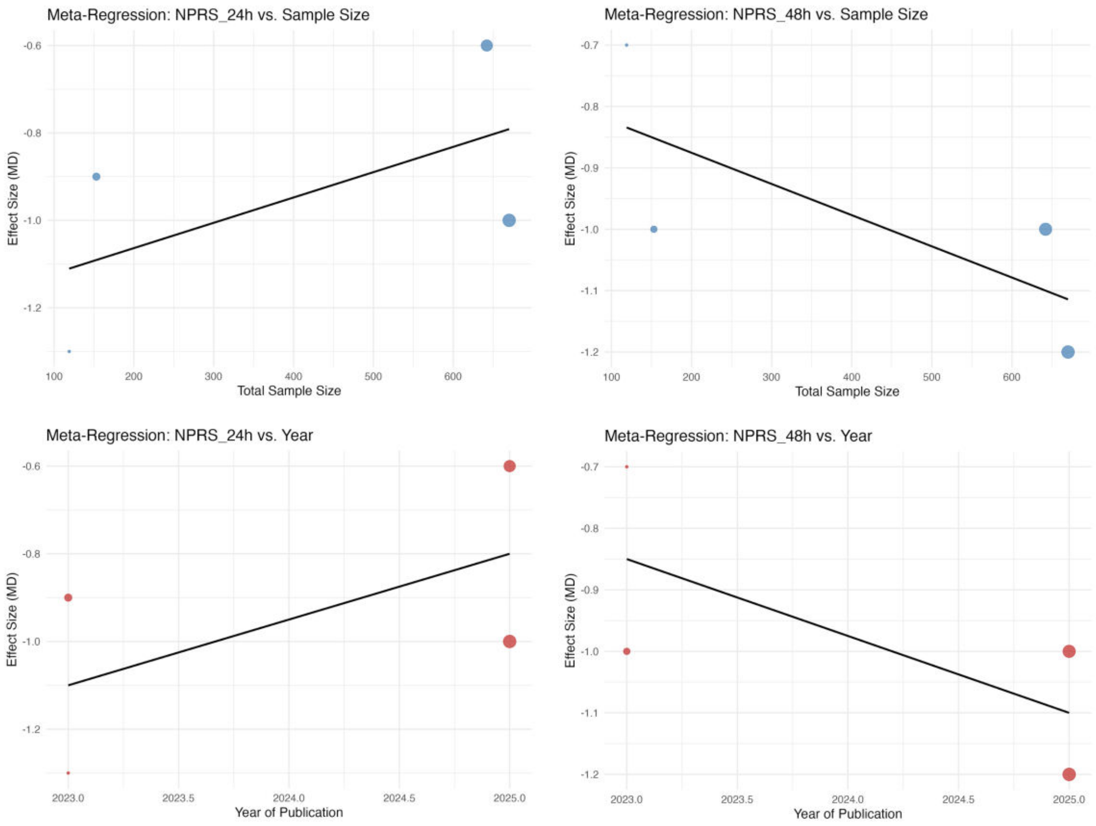

Figure S2 – Meta regression: bubble plot binary outcomes

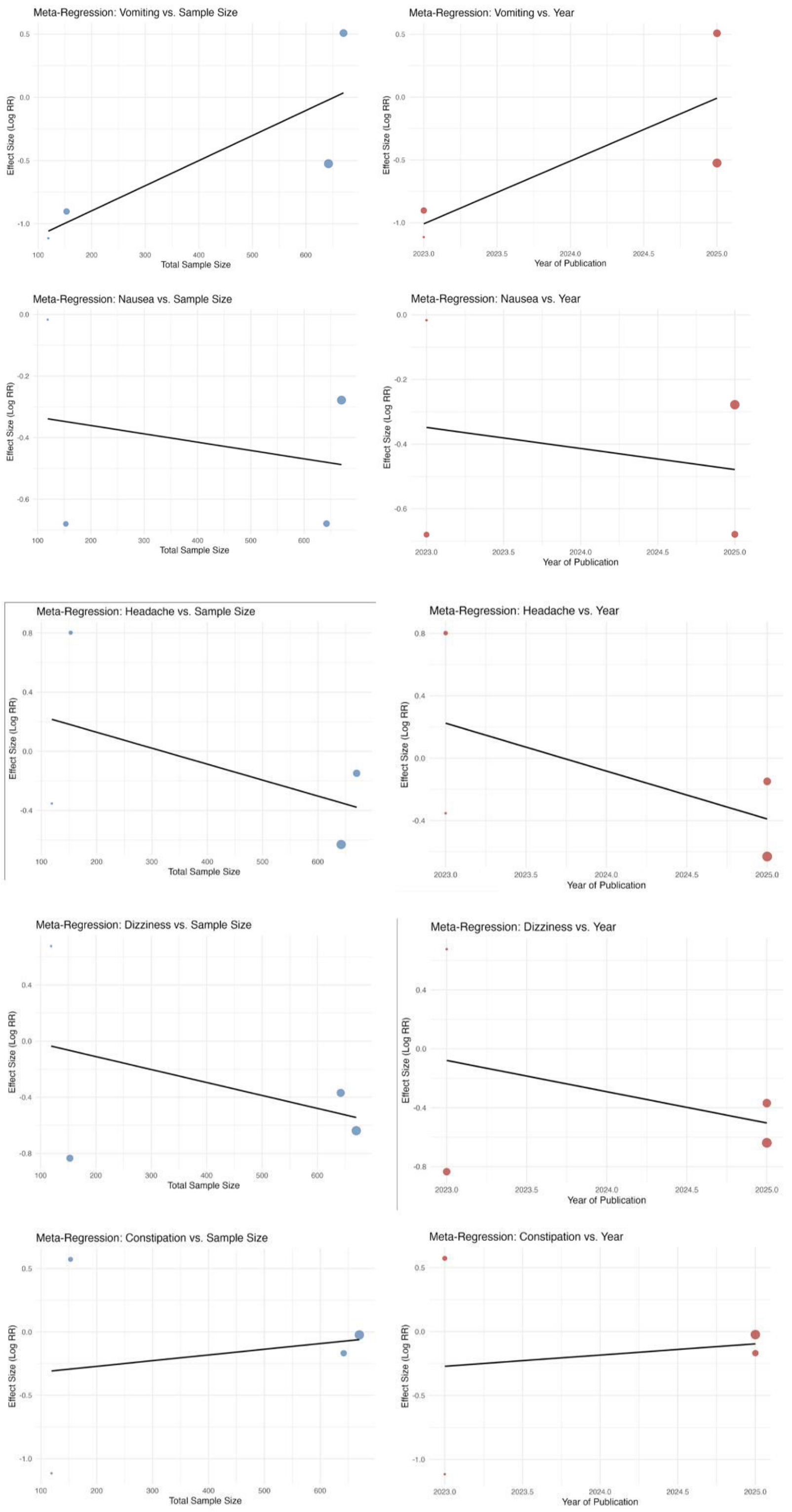

Figure S3 – Leave-one-out sensitivity analyses: primary outcome

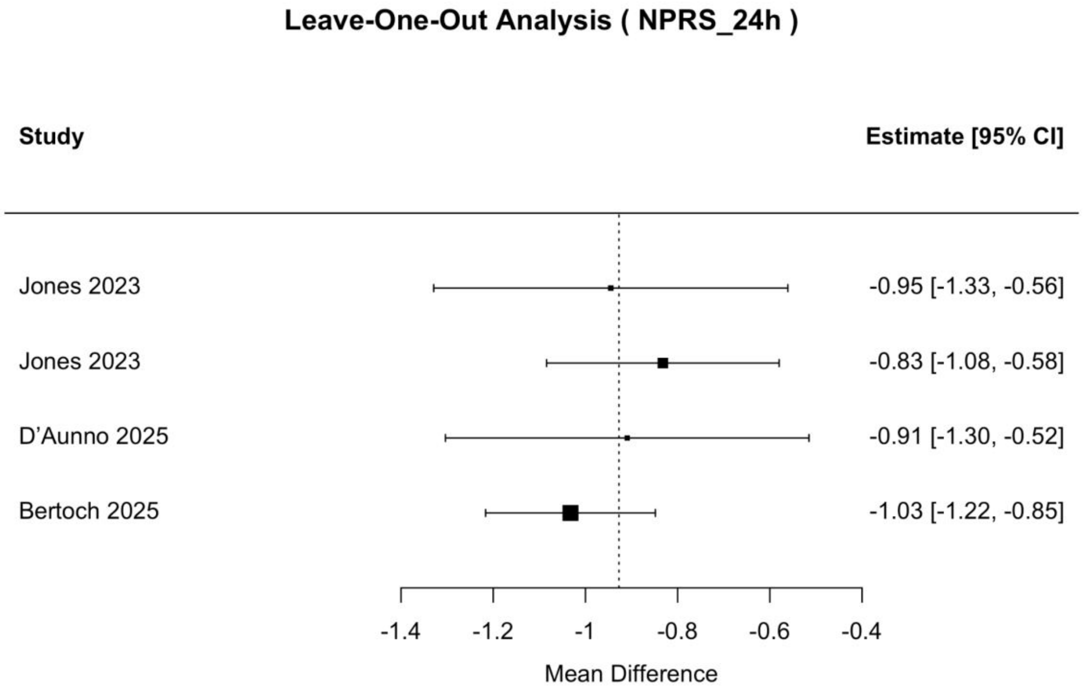

Figure S4 – Leave-one-out sensitivity analyses: continuous secondary outcomes

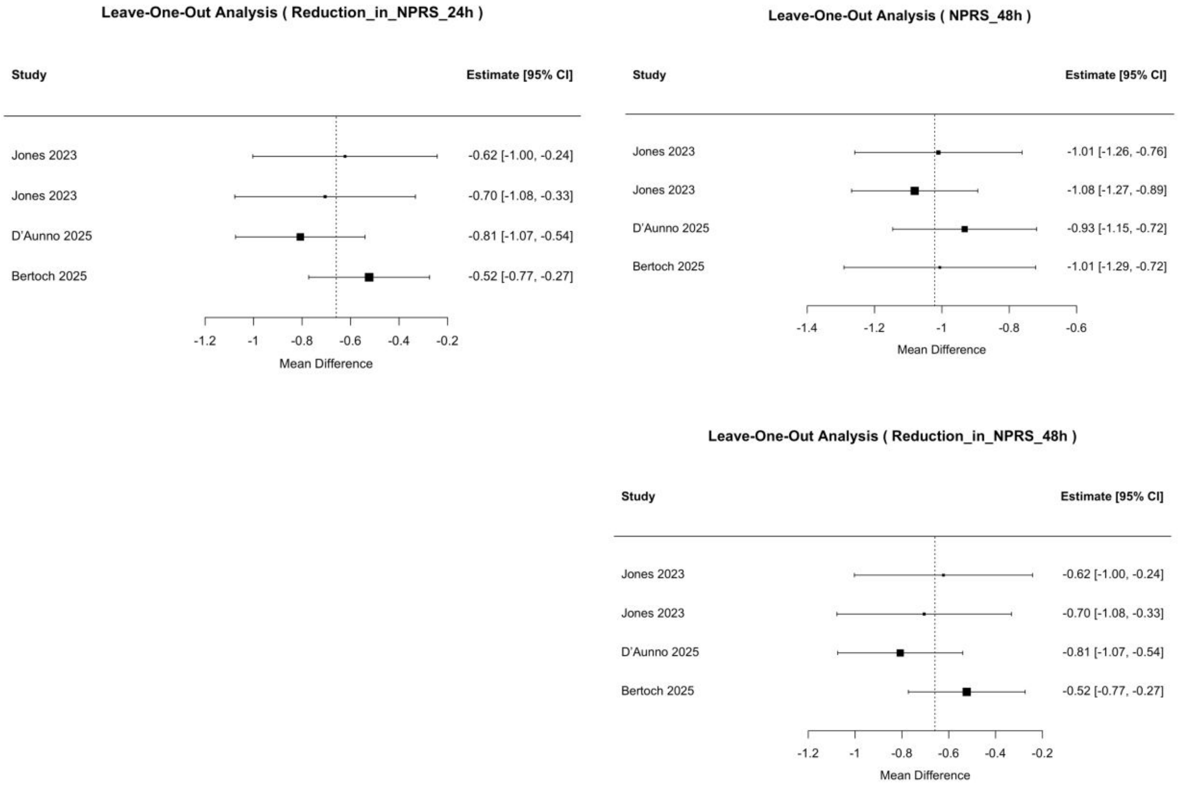

Figure S5 – Leave-one-out sensitivity analyses: binary outcomes

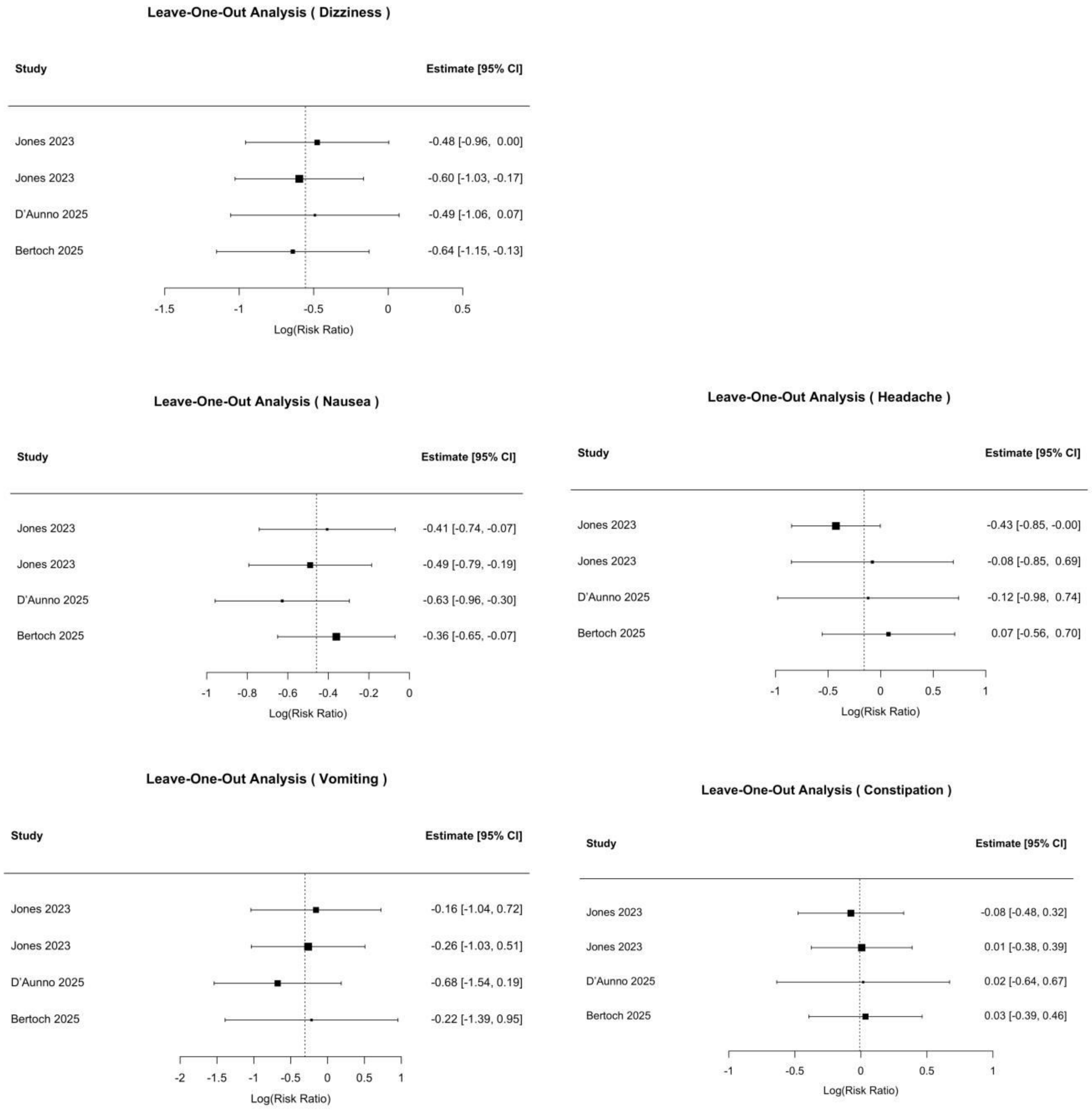

Figure S6 – Risk of bias assessment (Rob 2)

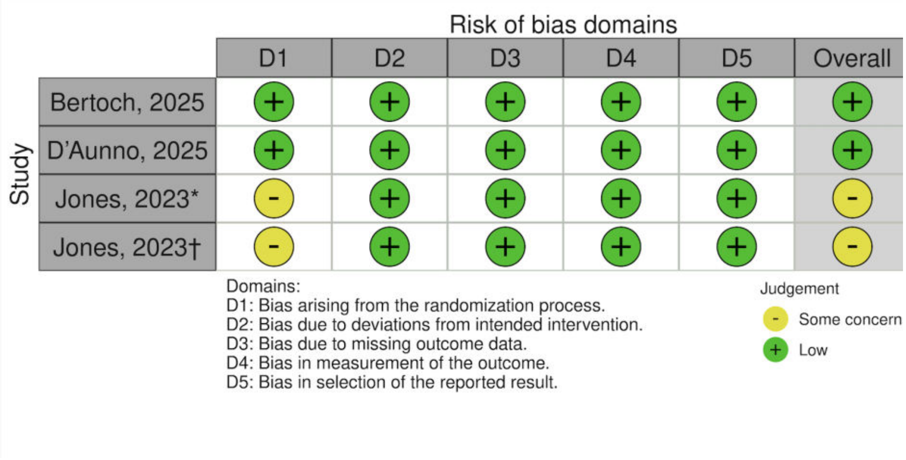

Figure S7 – Funnel plot: primary outcome

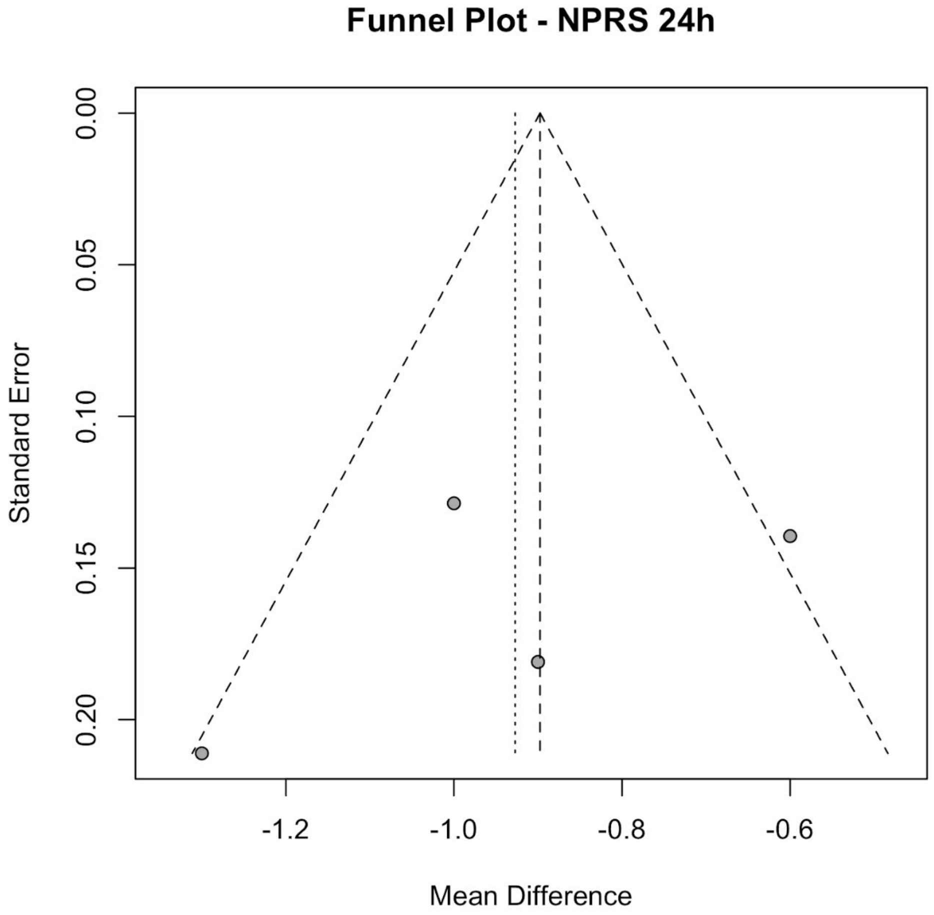

Figure S8 – Funnel plot: continuous secondary outcomes

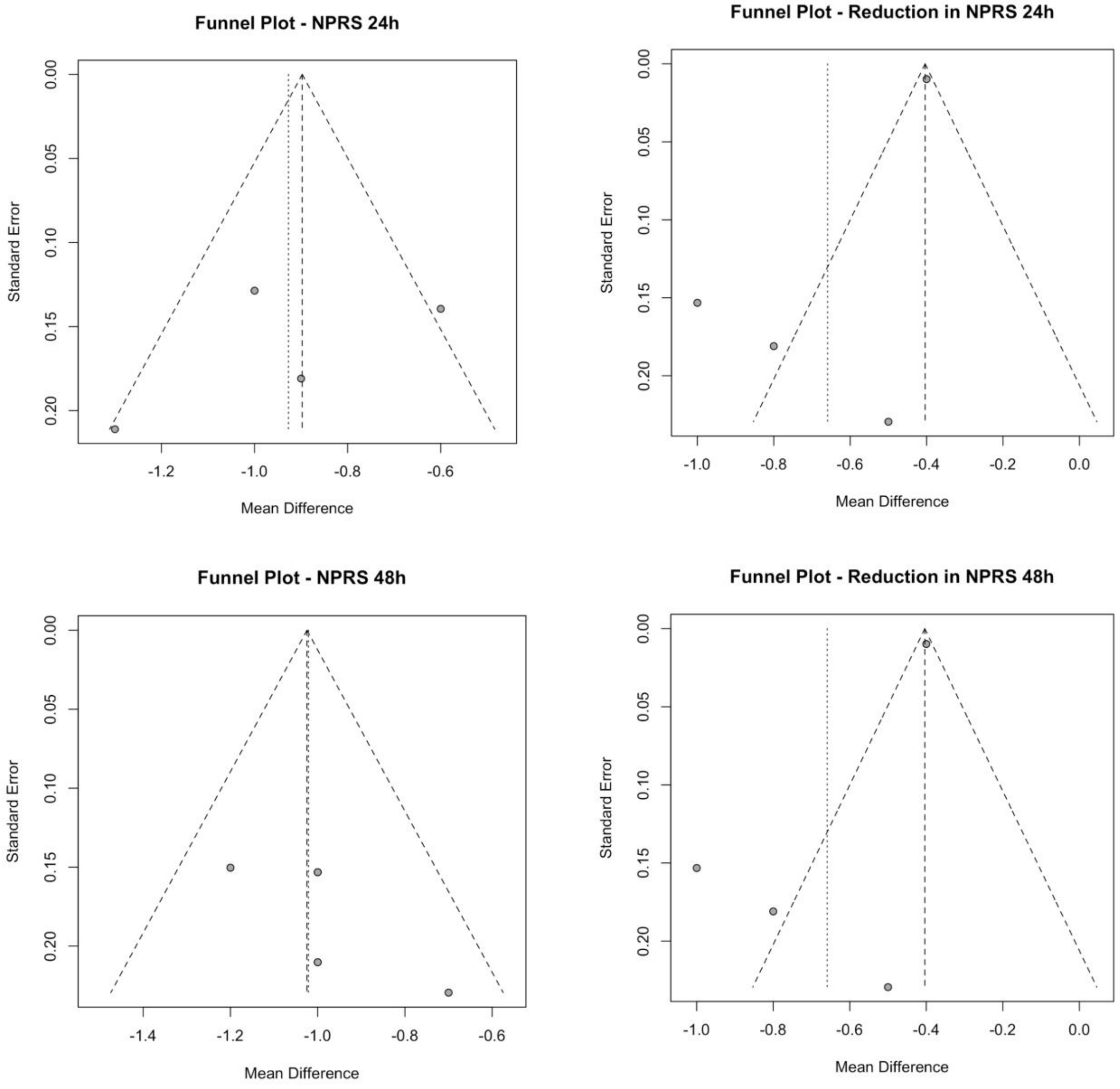

Figure S9 – Funnel plot: binary outcomes

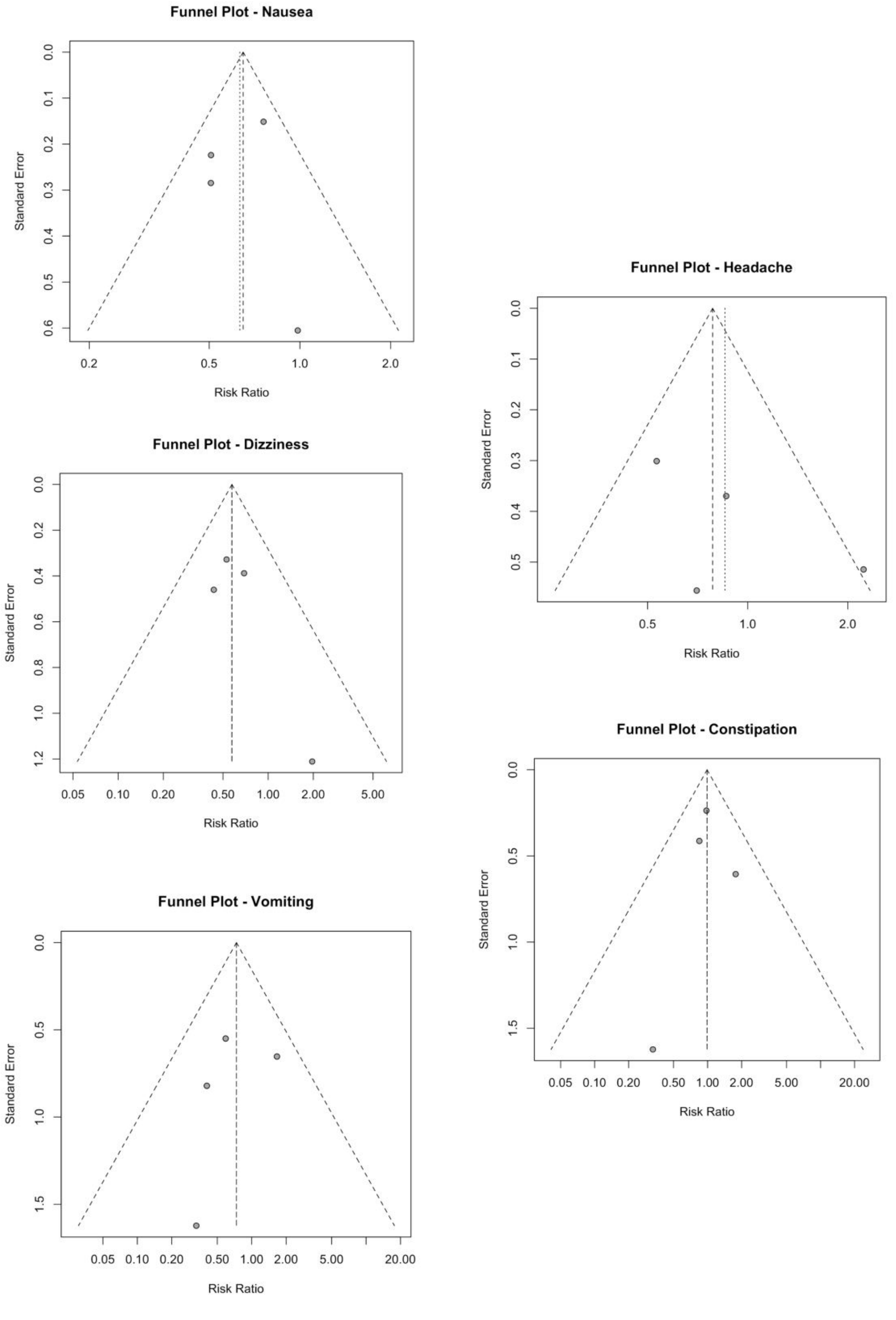

Supplement: Supplementary file 1 [file medi-105-e47877-s001.pdf]
